# Patient‐Led Research to Develop a Training Programme for Restoring Musical Joy in Cochlear Implant Recipients: A Reflexive Process Evaluation

**DOI:** 10.1111/hex.14133

**Published:** 2024-07-10

**Authors:** Marjo J. M. Maas, Joke Veltman, Philip J. van der Wees, Cilia Beijk, Wendy J. Huinck, Adinda Y. M. Groenhuis, Huib Versnel, Gertjan Schuiling, Alex E. Hoetink

**Affiliations:** ^1^ Radboudumc IQ Health Radboud University Nijmegen the Netherlands; ^2^ Department Allied Health Sciences HAN University of Applied Sciences Nijmegen the Netherlands; ^3^ Stichting Musi‐CI Breda the Netherlands; ^4^ Department of Otorhinolaryngology Radboudumc Nijmegen the Netherlands; ^5^ Donders Institute for Brain, Cognition and Behaviour Radboud University Nijmegen the Netherlands; ^6^ Department of Otorhinolaryngology and Head & Neck Surgery UMC Utrecht Utrecht the Netherlands; ^7^ School of Business and Economics, Management and Organisation VU University Amsterdam Amsterdam the Netherlands; ^8^ UMC Utrecht Brain Center Utrecht the Netherlands

**Keywords:** action research, cochlear implants, patient empowerment, patient involvement

## Abstract

**Background:**

The role of patients in healthcare research is slowly evolving, although patient roles in the research process are limited. This paper reports on a patient‐led research project aiming to develop a musical hearing training programme for patients with a cochlear implant (CI): the Musi‐CI programme. A CI is an inner ear prosthesis that allows people with severe hearing loss to hear. However, while speech can be understood, CI users cannot fully enjoy music or feel aversion to it. The Musi‐CI programme aims to reduce this music aversion to ultimately improve music enjoyment and social participation. The development of the Musi‐CI programme was supported by a consortium of professionals in CI rehabilitation and research.

The aim of this paper is to describe and evaluate the Musi‐CI programme development process and its impact on professional CI rehabilitation and research.

**Methods:**

Programme development was described using a 3‐layered process model of action research, distinguishing the CI user process, the healthcare professional process and the research process. To evaluate perceptions on the programme development process, consortium partners provided written comments and participated in a reflexive evaluation session that was video‐recorded. Reflexive evaluation aims for collective learning and strengthening collaboration among participants. Written comments and video data were analysed using template analysis.

**Results:**

The involvement of an expert by experience was perceived as challenging but rewarding for all consortium partners, opening up new perspectives on CI‐rehabilitation practice and research. Data analysis revealed two themes on the programme development process, professional space and acknowledgement, and two themes on the outcomes on CI rehabilitation and research: critical reflection and paradigm shift.

**Conclusion:**

Experts by experience represent a different knowledge domain that may contribute to change in rehabilitation and research.

**Patient or Public Contribution:**

The development of the programme was initiated by a professional musician and CI user who organized the funding, had a leading role throughout the research process, including the write‐up of the results, and co‐authored this paper.

**Abbreviations**
CI: cochlear implant
PAR: participatory action research

## 1 Introduction

Since the introduction of patient‐centred care, patients are increasingly involved in clinical decision‐making, quality improvement, training design, policy development and research [1]. For many years, the development and evaluation of healthcare services were dominated by healthcare professionals and researchers, missing the unique perspective of patients and hindering the implementation of research findings in the clinic [2]. More inclusive research approaches, allowing for ‘the patient voice’ in the research process, may contribute to quality improvement in clinical practice [3].

Granting patients an active role in research is not self‐evident. Patients may have rich experiential knowledge, but their voice is not automatically considered as a valid source of information from the perspective of health professionals and researchers [4]. Patients may not be identified as credible experts, and health professionals and researchers may unconsciously hold on to traditional knowledge hierarchies [5, 6]. Patient (experiential) knowledge is personal, often implicit and context‐bound. Expert knowledge is considered more objective and generalizable and therefore assumed to be less biased [7].

Patient involvement in the health research process has modestly evolved over the past decade [8], but patient roles in research are limited. A scoping review of McCarron et al. showed that the most common role for patient partners was a member of the research team or advisory group [9]. A new step in patient involvement is patient‐led research, in which patients actually lead the research project and collaborate with healthcare professionals, researchers and other stakeholders, often forming partnerships to address issues that directly affect their lives [10]. In conventional medical research, researchers decide what outcomes matter. In patient‐led research, patients decide what outcomes matter, and these outcomes may differ from conventional research outcomes [7]. Patient‐led research is relatively new, and reports on the process and outcomes are limited to specific conditions such as dementia, diabetes, arthritis, ALS and Covid‐19 [10, 11, 12, 13, 14, 15].

In this paper, we report on the evaluation of an innovative patient‐led research project aiming to develop a musical hearing training programme for patients with a CI: the Musi‐CI programme. A CI is an inner ear prosthesis that allows people with severe hearing loss to hear. However, while speech can be understood, CI users cannot fully enjoy music or feel aversion to it. Despite research showing that 90% of CI users would like to have music rehabilitation after cochlear implantation [16], it is still not integrated into standard CI rehabilitation. In addition, there is accumulating evidence that CI users may benefit from the cross‐modal plasticity of the brain and multisensory integration when training musical abilities [17]. Clinicians, however, are hesitant in promises regarding music perception with CI due to the neuroprosthetic nature of the CI affecting music pitch, timbre and dynamics perception. This paper shows how patient‐led research may contribute to change in standard rehabilitation practice and research.

The innovative character of this study is twofold: [1] the Musi‐CI programme aims to enhance music enjoyment and societal participation, while regular CI rehabilitation aims for understanding speech, recognition of environmental sounds and communication [2]; a CI user (former patient) initiates the research, organizes the funding and has a leading role in the collaboration between CI users, CI rehabilitation therapists and researchers.

The aim of this paper is to describe and evaluate the development process of the Musi‐CI programme and the impact of user involvement on professional CI rehabilitation and research. We introduce this paper with the following case.

## 2 The Case

J.V. is a musician and music teacher who gradually became deaf. In the next section, J.V. shares her experiences.

“I received a CI in 2013. Being motivated by **my** desire to resume my professional activities, **I** trained myself in listening to music with the CI’. When **I** visited my medical CI specialist, he emphasized that hearing music with CI was impossible. Avoiding overly high performance expectations is common practice in CI rehabilitation as pre‐operative expectations may affect patient outcomes [13]. Despite this discouragement, I did not lower my ambitions. I continued deliberate practice and in addition, I completed a master's degree on music to have a better understanding of how music is processed in the brain. After comprehensive self‐training, I gradually rediscovered the pleasure of listening to music and resumed my teaching activities. During my latest visit to my CI specialist, I shared my ability to play the piano again and my improved musical enjoyment. However, my doctor did not seem convinced and assured me that learning a new piece would be beyond my capabilities. Nevertheless, I started studying a new piece: Beethoven's 3^rd^ piano trio, the next day.”

J.V. passionately wished to share her musical experiences for the benefit of other CI users. Strengthened by her belief that CI users wish for and deserve a more comprehensive CI rehabilitation, including music perception, she developed a musical listening training programme for CI users based on her experiences as a professional piano teacher and personal experiences as a CI user. This programme aimed to enhance music enjoyment and social participation of CI users. She approached university medical centres in the Netherlands for research on her training programme. Although some CI rehabilitation specialists and researchers were impressed by the demonstrated musical abilities of J.V. and supportive of the idea to integrate music into regular CI rehabilitation, they did not decide to put the topic on the research agenda of their institutes. Finally, J.V. searched for funding herself.

J.V. founded the Musi‐CI foundation and brought together a team of different healthcare disciplines involved with CI rehabilitation from two academic hospitals, the Musi‐CI consortium (*n* = 7), to support her. From the first hospital: C.B. (speech and language pathologist), W.H. (speech and language scientist), from the second hospital: A.G. (speech and language pathologist), H.V. (CI and hearing scientist), A.H. (medical physics expert audiology and scientist) and the Musi‐CI foundation. M.M., the first author of this paper (allied health and education scientist), designed and navigated the research project as an action researcher. All consortium partners volunteered to participate, and shared their love for music and their willingness to explore new pathways in CI rehabilitation. J.V. applied for a grant of the funding programme ‘ZonMw Voor Elkaar’ supported by the consortium. The aim of this call for grants was to finance projects of organizations whose core task is to defend the interests of people with a chronic illness and support the use of experiential knowledge and expertise. Lay persons are allowed to apply for this grant. In July 2019, the Musi‐CI foundation received a grant for the development and evaluation of a musical hearing training programme. The consortium can be considered as a result‐accountable team that worked on behalf of the Musi‐CI foundation. A participatory action research (PAR) approach was chosen to develop and evaluate the Musi‐CI programme and to write a handbook for CI‐rehabilitation professionals involving all consortium partners and CI users (training participants) as programme developers. PAR is a collaborative approach to research that involves active participation from both researchers and the individuals or communities directly affected by the research topic. PAR emphasizes a cyclical process of (1) Plan, (2) Act, (3) Observe and (4) Reflect with the goal of empowering participants to drive positive changes in healthcare practices and policies [3]. The PAR approach was new to the consortium partners. J.V. was educated by M.M. in the PAR methodology.

## 3 The Musi‐CI Programme Development Process

**Figure 1:**
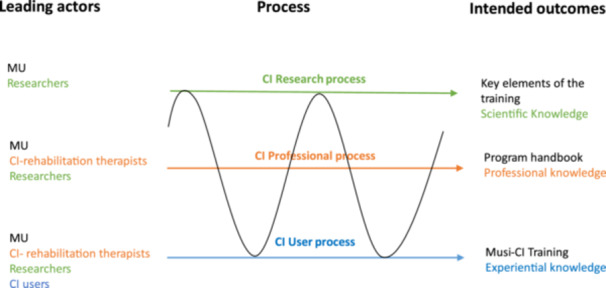
Three processes in action research, according to Schuiling and Kiewiet [18].

The consortium developed the Musi‐CI programme in three PAR cycles of plan, act, observe and reflect in close collaboration with CI users. The first cycle started in autumn 2019 and the third cycle was finished in fall 2020. To describe and evaluate how the consortium collaborated to stepwise improve the Musi‐CI programme, we used a 3‐layered process model of action research (Figure 1) that distinguishes the CI research process, the CI professional rehabilitation process and the CI user process [18]. The black line between the layers presents the movements of J.V., showing that she was involved in all layers of the programme development process in different roles: CI user and expert by experience, professional music teacher and researcher. J.V. was empowered by M.M. to take up the researcher role.

### 3.1 The Research Process

The upper layer in Figure 1 shows the research process exploring the key elements of the training programme and the underlying programme theory, resulting in a theoretical framework (scientific knowledge). First, a draft training programme was designed based on the experiences of J.V. Additionally, a preliminary programme theory was developed by exploring the implicit and explicit assumptions of J.V. as a music teacher and CI user [19, 20]. J.V. was challenged by M.M. to step out of her ‘patient’ role and to take on a helicopter perspective on her experiences as a CI user. Her ‘lived’ knowledge as a CI user and professional knowledge as a musician were explored by critically questioning J.V. about what works, why and under what circumstances. This knowledge was compared to the existing literature on adult learning and CI rehabilitation. A draft theoretical framework and a draft professional framework were developed containing the first key elements of the Musi‐CI programme. These frameworks were stepwise improved and refined according to the input of CI users, CI rehabilitation therapists and researchers.

### 3.2 The Professional Process

The second layer in Figure 1 shows the professional development process aiming for the development of a handbook available for CI rehabilitation therapists who intend to integrate music into CI rehabilitation. Observations of training sessions by CI rehabilitation therapists (notes) were discussed and compared to their professional knowledge and the knowledge of J.V. Results were used to inform the research process and to stepwise develop a handbook for professionals.

### 3.3 The CI User Process

The bottom layer represents the experiences of CI users with the Musi‐CI programme that ultimately resulted in improved music enjoyment and social participation. J.V. challenged CI users to engage with music, helping them to discover new ways of listening by using her unique experiential and professional knowledge. Experiences of participants were evaluated on ‘what works and what does not’, and results were used to inform the professional and research process and to stepwise improve the programme.

The consortium wrote a report for the funding organization and a scientific paper on the Musi‐CI programme that was published in 2023, showing promising results [21]. The research project was awarded by the funding organization ZonMw in May 2022 for its impact on the field [22].

## 4 Reflexive Evaluation of the Programme Development Process and Its Impact on Professional CI Rehabilitation and Research

When the project was finalized and the results were published, all consortium partners (including J.V.) reflected on the programme development process and the perceived impact of this research project on CI rehabilitation and research.

## 5 Methods

Reflexive evaluation is part of the PAR methodology aiming for collective learning and strengthening collaboration among participants. The evaluation involves both looking back and forward to inform future research projects. Evaluator and evaluatees come together for collective evaluation, interpretation of the findings and to identify areas for potential change [3, 23].

### 5.1 Data Sampling

During biweekly online consortium meetings, the evaluation plan was introduced by M.M. All consortium partners consented to participate. They responded individually in writing—to avoid mutual influencing—to an open question sent by email by M.M.: ‘Would you please describe your experiences with this research project and the impact on CI rehabilitation practice and research?’ Subsequently, an online 1.5 h video session was conducted and facilitated by A.H., an experienced discussion leader. A.H. invited each participant to share their written comments with the group and to respond to probing questions. Subsequently, A.H. invited the group for a rapid evaluation data analysis to identify the main topics of the evaluation results.

### 5.2 Data Analysis

An in‐depth analysis was conducted by M.M. and an external researcher who was not a member of the consortium GJ.S. to enhance the reflexivity and trustworthiness of the findings. Written comments and video data were triangulated and entered by M.M. into Atlas.ti qualitative analysis software (https://atlasti.com/what-s-new-in-atlas-ti-23ef) and coded using Template Analyses guidelines allowing a priori codes (prior identified key topics by the consortium) and emerging codes from the written and video data sources [24]. Coded video data were transcribed verbatim. By iteratively comparing and discussing codes, comparing codes to the existing literature and merging codes into categories, overarching themes were identified and described. A member‐checking procedure was conducted by sharing the data analysis report with all consortium partners and modifying the results according to their feedback. They all agreed to this publication. We adhered to COREQ consolidated criteria for reporting qualitative research [25].

## 6 Results

All invited consortium partners (*n* = 6) returned their written responses via email to M.M. The number of words varied from 212 to 420. They all participated in the evaluation session.

The analysis of the data unveiled two themes on the programme development process: professional space and acknowledgement, and two themes on the impact of CI user involvement on CI rehabilitation and research: critical reflection and paradigm shift. In the next section, themes are described and illustrated by quotes stemming from written data or video data.

### 6.1 Perceptions of the Programme Development Process

Although the consortium was effective in achieving its intended outcomes, as published earlier [21], the programme development process was at times perceived as tense. Participants provided critical but constructive comments on the programme development process to learn from each other and strengthen collaboration for the planned future projects involving J.V.

#### 6.1.1 Confusion About Professional Space

Despite the unique knowledge of J.V. as an expert by experience, a professional musician and a self‐educated trainer of the Musi‐CI programme, she was not a healthcare professional or researcher. She belonged to the consortium but not to the rehabilitation teams within the academic setting. Except for her experiential knowledge, J.V. did not feel fully accepted as a sparring partner among CI professionals and researchers.

JV Yes, I have noticed that it is a long way for an outsider, what is my role, how much [professional] space do I get, sometimes I get the feeling that I am being put back in the box of the expert by experience, while I feel like a professional as well, but don't always feel seen that way.

(written comment)

Although the aim of the Musi‐CI project was clearly defined, boundaries of professional expertise were not set and expectations of professional activities were not discussed in advance. The purposes of clinical CI rehabilitation (learning to recognize environmental sounds and to understand speech and communicate) and Musi CI rehabilitation (learning to listen and enjoy music) became intertwined. Crossing professional domains caused confusion within the consortium about the true aim of the Musi‐CI programme and the opportunity to address this issue was missed.

AG (therapist). I think that in this project we should only focus on music listening and music enjoyment and not understanding of speech, because I think this is the territory of the speech and language therapists, perhaps even the territory of the entire professional CI rehabilitation team. I really want to advocate that there should be no overlap with the things we do in regular CI‐rehabilitation and in the Musi‐CI project.

(video comment)

JV It came to my attention that my presence as a patient‐professional and patient‐researcher might have been perceived as threatening [to CI rehabilitation therapists]….we somehow didn't succeed in openly discussing this issue [….].

(video comment)

#### 6.1.2 Little Acknowledgement

All consortium partners were involved in providing input and feedback on the Musi‐CI programme and the Musi‐CI handbook. However, different perspectives on the added value of the music‐CI training for CI‐rehabilitation were not explicitly addressed during the project, and consortium partners did not all feel sufficiently acknowledged for their expertise and innovative ideas.

CB (therapist). I noticed that… I had a lot of ideas [to improve the program], but I wondered where my ideas had gone… and where my name would end up. I would appreciate my input being acknowledged.

(video comment)

### 6.2 Perceptions of the Programme Development Outcomes

Despite the challenges in the collaborative process, or perhaps because of those challenges, the research project has yielded interesting results for all stakeholders in CI rehabilitation. All consortium partners were open to new perspectives on CI rehabilitation and demonstrated a willingness to explore what was previously considered impossible: enjoying music.

#### 6.2.1 Trigger for Change

The research project allowed the CI rehabilitation therapist to observe and discuss the Musi‐CI programme as conducted by J.V. Although the Musi‐CI programme was not immediately embraced by all CI rehabilitation therapists, it triggered reflection on daily CI rehabilitation and opened doors for innovation inside and outside the context of academic hospitals.

AG (therapist): ‘If you always do what you've always done, you always get what you've always got. In my training as a speech and language therapist I learned that reflection is crucial…’. ‘I could not imagine it would be possible for so many CI users to enjoy music again. I thought the training might only work for MU because she is a top performer with regard to speech perception and as a musician. Since I have seen it work for many CI users, I have made a significant shift in my judgement’.

(written comment)

WH (researcher): The Musi‐CI project has contributed to awareness of rehabilitation therapists and CI users as well as care managers regarding the added value of music for CI users. Applying the practical knowledge developed during this project was useful for both clinical practice and research.

(video comment)

CI rehabilitation therapists were motivated to bring music into their regular CI rehabilitation practice, building on the findings of this research project. J.V. and the participating CI professionals could be considered game changers in this respect.

CB (therapist) Thanks to JV…, we have taken up music again. In the past, we tried to put flesh on the bones of music within CI rehabilitation. We knew from patient evaluations that there was a demand, but we did not feel that our way of working contributed to the improvement of the music experience. Because JV, as an expert by experience, emphasized the importance of music for CI users, we started to take up the topic again.

(written comment)

#### 6.2.2 Paradigm Shift

The researchers involved were inspired to expand their field of research: from knowledge grounded in scientific evidence (the positivist paradigm) to knowledge constructed from multiple perspectives (interpretative paradigm) [20]. This shift also applied to the research methodology and the outcomes used: from quantitative methods and clinical outcomes to mixed methods and self‐reported outcomes [2, 7].

AH (researcher) I also gained new insights. For example, JV told us [.] that we as CI specialists, we are primarily focused on auditory performance(s) such as ‘recognizing' melodies or musical instruments. She taught us that, for patients with a CI, we can better focus on ‘exploring’ music. CI users can find out for themselves what music and what instrumental sounds they like. This can be very different to what they enjoyed before they were implanted. In other words, the goal is not to make music sound how it ought to, because that is usually not feasible. It is about being able to enjoy music and instruments again in your own particular way.

(written comment)

HV (researcher) Music is on the bucket list of CI researchers since I work in this field (twenty years). A researcher wants to demonstrate an effect of a training, whatever this effect may be. But we should not be blinded by it. It is possible that a CI user is happy with a training that is not evidence‐based. When I met participants during a Musi‐CI program session or at an interview I saw happy faces. Therefore, it is very good that the Musi‐CI project has so far focused on exploring the possibilities of music to enrich the CI rehabilitation program and that we were patient regarding the measurement of effect. I believe that our strength is in building a strong Musi‐CI program that may yield larger effects, which are then easier to measure.

(video comment)

AH What I would like to add to what has been said is that it has become even clearer to me that the outcome measures that healthcare professionals and researchers use can be very different from the outcome measures that are important to CI users. The fact that an expert by experience (who in our case is also a professional) took the lead, ensured that the second type of outcome measure had top priority [music enjoyment]…. It is not so much about being able to recognize music (the traditional ‘hard’ outcome measure for effectiveness), but about being able to enjoy a melody even though it does not sound like it used to and is not recognizable.

(video comment)

## 7 Discussion

The aim of this paper was to describe and evaluate the Musi‐CI programme development process and the impact on CI rehabilitation and research. Programme development was described using a 3‐layered process model of action research, distinguishing the CI user process, the healthcare professional process and the research process. The involvement of an experienced CI user in all layers of the programme development process was challenging but key to the positive outcomes. Interaction with J.V. resulted in changes in the mindsets of CI rehabilitation therapists and researchers. The role of J.V. can be viewed as ‘transformative’, as outlined by Greenhalgh in her blog on patient‐led research, which underscores the significance of addressing outcomes that matter to patients [7]. However, the results also show that J.V. was insecure in performing the different roles as explained in the three processes model and needed the empowerment and guidance of M.M. to take the lead and to steer the project towards its intended outcomes.

Although music training programmes have been offered to CI users in the past [26], the innovative strength of this project lies in its action research approach and the unique knowledge of J.V., which enabled tailoring the programme to the individual needs, preferences and capabilities of CI users. J.V., along with the participating CI users, provided a fresh perspective on CI rehabilitation, expanding its benefits beyond speech understanding to enhancing music appreciation and shifting from regaining lost capabilities to discovering new capabilities, a shift that is in line with the principles of positive health [27] and the values of person‐centred care. So far, J.V.'s involvement in designing a music training programme has been successful in opening new horizons and instigating positive changes in CI rehabilitation. However, J.V. cannot be compared to the average CI user. Her exceptional qualities can be seen as a limitation of this study regarding the transferability of the findings. However, the literature demonstrates the necessity of competent patients to bring about change in routine practice. A scoping review of Frisch et al. on successful patient involvement in research resulted in a competency statement for both patients and researchers [28]. The two critical elements for patients were ‘lived’ experience in the condition being studied and an interest in participating in research. Patient competencies were related to research knowledge and skills, cultural competence and active participation. J.V. was a competent patient.

Looking at the critical competencies of researchers, Frisch et al. mention the skill to identify and discuss areas of potential tension and resistance within the team. The perceptions of the consortium partners on the programme development process revealed shortcomings in communication and tensions in the professional boundaries. The CI rehabilitation therapists accepted J.V. as an expert by experience but were reluctant in allowing J.V. to interfere with their professional domain and rehabilitation goals, causing frustrations on both sides now and then. These tensions are well described in the PAR and patient involvement literature [4, 9, 29, 30]. The literature also shows that establishing a shared vision about the goals of patient engagement and respective roles is crucial, and honesty and trust are key to effective patient involvement in research [31]. We assume that timely reflection on this issue to appreciate the nature of the tensions and to better understand what happens in the professional boundary area would have strengthened the programme development process [32]. We may conclude that the fourth step in the PAR cycle—plan, act, observe and reflect—has not received sufficient attention.

Looking at the implications of this study for CI rehabilitation, the involvement of J.V. has strengthened the need to integrate music into a standard CI rehabilitation. Given that the inclusion of music training in hearing rehabilitation after cochlear implantation is a wish of 90% of CI users [33] and that the rationale of including it lies in the emerging concepts of cross‐modal plasticity and multisensory integration, it is crucial for clinicians and policymakers responsible for facilitating this integration to acknowledge the significance of music training to improve the social participation and the quality of life of CI users [16].

## 8 Strengths

Since the patient‐centred movement, the literature and insights on patient involvement have grown rapidly. However, guidance is limited to research projects involving patients in the researcher role. With this research project, we have started to bridge a gap in the literature on patient‐led research projects.

## 9 Limitations

In this research project, we focused on explicating the experiential and professional knowledge of J.V. to build a training programme that matches the needs, preferences and abilities of CI users. However, by focusing on the knowledge of J.V. only, we may have missed relevant input of CI rehabilitation experts. Although expert knowledge is assumed to be explicit, it often becomes internalized by experience. Explicating this expert knowledge might have contributed to a broader acknowledgement of the existing expertise within the consortium.

## 10 Future Research

To contribute to theory on the programme development of knowledge in the context of patient‐led research and to enhance the transferability of our findings, future studies describing comparable cases in different contexts are needed.

## 11 Conclusion

In this paper, we reported on the programme development process and outcomes of a patient‐led project aiming to add value to the lives of CI users. The 3‐layered process model on knowledge creation was helpful to describe the programme development process and its impact on CI rehabilitation and research. The results showed that patient expertise represents a different knowledge domain that may trigger change in clinical practice and research. However, patient empowerment to take up new roles and conscious reflection on the programme development process are needed.

## Author Contributions

**Marjo J.M. Maas:** conceptualization, investigation, writing–original draft, methodology, validation, formal analysis. **Joke Veltman:** writing–review and editing, conceptualization, investigation. **Philip J. van der Wees:** validation, writing–review and editing, supervision. **Cilia Beijk:** writing–review and editing. **Wendy J. Huinck:** writing–review and editing. **Adinda Y.M. Groenhuis:** writing–review and editing. **Huib Versnel:** writing–review and editing, writing–original draft. **Gertjan Schuiling:** conceptualization, writing–review and editing, supervision, formal analysis. **Alex E. Hoetink:** supervision, writing–review and editing.

## Conflicts of Interest

The authors declare no conflicts of interest.

## Data Availability

The data that support the findings of this study are available on request from the corresponding author, marjo.maas@radboudumc.nl. The data are not publicly available due to information that could compromise the privacy of research participants.

## References

[1] “Crossing the Quality Chasm: A New Health System for the 21st Century,” Institute of Medicine, 2001.

[2] T. Greenhalgh , *How to Implement Evidence‐Based Healthcare*, 1st ed. (Oxford: Wiley & Sons, 2018).

[3] T. Abma , S. Banks , and T. Cook , Participatory Research for Health and Social Well‐Being, 1st ed. (Switzerland: International Nature, 2019).

[4] C. Bergerum , J. Thor , K. Josefsson , and M. Wolmesjö , “How Might Patient Involvement in Healthcare Quality Improvement Efforts Work. A Realist Literature Review,” Health Expectations 22, no. 5 (2019): 952–964. 31044517 10.1111/hex.12900 PMC6803394

[5] M. Fricker , “Evolving Concepts of Epistemic Injustice,” in The Routledge Handbook of Epistemic Injustice, eds. I. J. Kidd , J. Medina , and G. Pohlhaus (Routledge, 2017), 53–60, https://eprints.whiterose.ac.uk/110047/3/Evolving%20Concepts%20of%20EI.

[6] M. Batalden , P. Batalden , P. Margolis , et al., “Coproduction of Healthcare Service,” BMJ Quality & Safety 25, no. 7 (2016): 509–517. 10.1136/bmjqs-2015-004315 PMC4941163 26376674

[7] T. Greenhalgh , “Towards an Institute for Patient‐Led Research,” BMJ, November 12, 2019, https://blogs.bmj.com/bmj/2019/11/12/trisha-greenhalgh-towards-an-institute-for-patient-led-research/.

[8] J. P. Domecq , G. Prutsky , T. Elraiyah , et al., “Patient Engagement in Research: A Systematic Review,” BMC Health Services Research 14 (2014): 89. 24568690 10.1186/1472-6963-14-89 PMC3938901

[9] T. L. McCarron , F. Clement , J. Rasiah , et al., “Patients as Partners in Health Research: A Scoping Review,” Health Expectations 24, no. 4 (2021): 1378–1390. 34153165 10.1111/hex.13272 PMC8369093

[10] L. B. Mader , T. Harris , S. Kläger , I. B. Wilkinson , and T. F. Hiemstra , “Inverting the Patient Involvement Paradigm: Defining Patient Led Research,” Research Involvement and Engagement 4 (2018): 21. 30002875 10.1186/s40900-018-0104-4 PMC6038253

[11] L. McCorkell , G. S. Assaf , H. E. Davis , H. Wei , and A. Wei Akrami , “Patient‐Led Research Collaborative: Embedding Patients in the Long COVID Narrative,” PAIN Reports 6, no. 1 (2021): e913. 33987484 10.1097/PR9.0000000000000913 PMC8112577

[12] D. Berkovic , I. Ackerman , and R. Buchbinder , “Patient‐Led Research in Rheumatology: The Way Forward?” Lancet Rheumatology 5, no. 4 (2023): e180. 10.1016/S2665-9913(23)00061-9 38251519

[13] A. Aboaja , O. Atewogboye , M. Arslan , L. Parry‐Newton , and L. Wilson , “A Feasibility Evaluation of Discovery Group: Determining the Acceptability and Potential Outcomes of a Patient‐Led Research Group in a Secure Mental Health Inpatient Setting,” Research Involvement and Engagement 7, no. 1 (2021): 67. 34563267 10.1186/s40900-021-00310-0 PMC8465701

[14] J. Kempner and J. Bailey , “Collective Self‐Experimentation in Patient‐Led Research: How Online Health Communities Foster Innovation,” Social Science & Medicine 238 (2019): 112366. 31345612 10.1016/j.socscimed.2019.112366

[15] M. I. Stefanou and I. Amygdalos , “Patient‐Led Research in Amyotrophic Lateral Sclerosis: Quo Vadis?” Amyotrophic Lateral Sclerosis and Frontotemporal Degeneration 16, no. 5–6 (2015): 418–422. 25800785 10.3109/21678421.2015.1013968

[16] A. Frosolini , G. Badin , F. Sorrentino , et al., “Application of Patient Reported Outcome Measures in Cochlear Implant Patients: Implications for the Design of Specific Rehabilitation Programs,” Sensors 22, no. 22 (2022): 8770. 36433364 10.3390/s22228770 PMC9698641

[17] R. A. Stevenson , S. W. Sheffield , I. M. Butera , R. H. Gifford , and M. T. Wallace , “Multisensory Integration in Cochlear Implant Recipients,” Ear & Hearing 38, no. 5 (2017): 521–538. 28399064 10.1097/AUD.0000000000000435 PMC5570631

[18] G. Schuiling and D. J. Kiewiet , “Action Research: Intertwining Three Exploratory Processes to Meet the Competing Demands of Rigour and Relevance,” Electronic Journal of Business Research Methods 14, no. 2 (2016): 111–124.

[19] J. Pols , “Knowing Patients,” Science, Technology, & Human Values 39, no. 1 (2013): 73–97.

[20] A. Kothari , D. Rudman , M. Dobbins , M. Rouse , S. Sibbald , and N. Edwards , “The Use of Tacit and Explicit Knowledge in Public Health: A Qualitative Study,” Implementation Science 7, no. 7 (2012): 20. 22433980 10.1186/1748-5908-7-20 PMC3325865

[21] J. Veltman , M. J. M. Maas , C. Beijk , et al., “Development of the Musi‐CI Training, a Musical Listening Training for Cochlear Implant Users: A Participatory Action Research Approach,” Trends in Hearing 27 (2023): 23312165231198368. 37697865 10.1177/23312165231198368 PMC10496489

[22] “Parel voor het project Musi‐CI: ZonMw,” 2022, https://www.zonmw.nl/nl/actueel/parelprojecten/parel-voor-het-project-musi-ci/.

[23] S. van Veen , L. Verwoerd , and B. Regeer , Characteristics of Reflexive Evaluation—A Literature Review (Amsterdam: Vrije Universiteit Amsterdam, 2016).

[24] N. King , C. Cassel , and G. Symon , Using Templates in the Thematic Analysis of Texts. Essential Guide to Qualitative Methods in Organizational Research, 1 (London: Sage Publications, 2004), 256–240.

[25] K. J. Du , G. S. Li , K. Zhang , Y. Lin , F. Yang , and K. Hannes , “COREQ (Consolidated Criteria for Reporting Qualitative Studies),” Annals of Translational Medicine 10, no. 19 (2022): 1073. 36330400 10.21037/atm-2022-23 PMC9622469

[26] N. F. A. Shukor , J. Lee , Y. J. Seo , and W. Han , “Efficacy of Music Training in Hearing Aid and Cochlear Implant Users: A Systematic Review and Meta‐Analysis,” Clinical and Experimental Otorhinolaryngology 14, no. 1 (2021): 15–28. 32646208 10.21053/ceo.2020.00101 PMC7904420

[27] J. Huber , “How Should We Define Health,” British Medical Journal 343 (2011): 343.

[28] N. Frisch , P. Atherton , M. M. Doyle‐Waters , et al., “Patient‐Oriented Research Competencies in Health (PORCH) for Researchers, Patients, Healthcare Providers, and Decision‐Makers: Results of a Scoping Review,” Research Involvement and Engagement 6 (2020): 4. 32055415 10.1186/s40900-020-0180-0 PMC7011284

[29] C. A. Chew‐Graham , “Patient Involvement in Research—Participants or Collaborators?” Health Expectations 20, no. 3 (2017): 369–370. 28514520 10.1111/hex.12578 PMC5433541

[30] T. A. Abma , “Dialogue and Deliberation: New Approaches to Including Patients in Setting Health and Healthcare Research Agendas,” Action Research 17, no. 4 (2018): 429–450.

[31] M. L. P. MacLeod , J. Leese , L. Garraway , et al., “Engaging With Patients in Research on Knowledge Translation/Implementation Science Methods: A Self Study,” Research Involvement and Engagement 8, no. 1 (2022): 41. 35941661 10.1186/s40900-022-00375-5 PMC9358643

[32] G. Schuiling and H. Vermaak , “Four Contexts of Action Research: Crossing Boundaries for Productive Interplay,” International Journal of Action Research 13, no. 1 (2017): 5–23.

[33] K. Gfeller , V. Driscoll , and A. Schwalje , “Adult Cochlear Implant Recipients' Perspectives on Experiences With Music in Everyday Life: A Multifaceted and Dynamic Phenomenon,” Frontiers in Neuroscience 13 (2019): 1229. 31824240 10.3389/fnins.2019.01229 PMC6882382

